# Triage effectiveness: a framework for quantifying the effect of emergency triage prioritization

**DOI:** 10.1186/s12874-026-02918-w

**Published:** 2026-06-19

**Authors:** André Johansson, Viktoria Fodor, Jakob Lundager Forberg, Anna Ekwall, Ulf Ekelund

**Affiliations:** 1 Department of Clinical Sciences, Faculty of Medicine, Lund University, Lund, Sweden; 2 KTH Royal Institute of Technology, School of Electrical Engineering and Computer Science, Stockholm, Sweden; 3 Department of Emergency Medicine, Helsingborg Hospital, Lund University, Helsingborg, Sweden; 4 Department of Health Sciences, Faculty of Medicine, Lund University, Lund, Sweden

**Keywords:** Triage Effectiveness, Priority Triage, Priority Queues, Emergency Care, Performance Metrics, Waiting Time Analysis, Patient Prioritization, Quality Indicators, Queueing Theory

## Abstract

**Background:**

Emergency departments use triage to identify time-critical patients and reduce waiting times by assigning higher priority. However, no existing method directly measures this core clinical function. We developed and validated Triage Effectiveness (TE) as a framework for quantifying how well triage systems reduce waiting times for time-critical patients.

**Methods:**

Using data from 463,209 visits across eight emergency departments, we developed TE as a scale where 0% equals a first-come-first-serve strategy (no triage) and 100% equals perfect prioritization. The framework includes two complementary measures: 1. Waiting Time-based TE (WTE) measuring actual waiting time reduction, where 100% represents zero waiting time, and 2. Rank-based TE (RTE) measuring queue position improvement where 100% represents placement first in queue. Both can be calculated as theoretical TE (based only on priorities) and observed TE (actual clinical performance). We validated WTE and RTE calculations using analytical queueing models adapted for classification uncertainty and assessed transferability across hospitals.

**Results:**

Queueing theory validation showed strong alignment with both WTE and RTE calculations, with RTE demonstrating greater robustness and less sensitivity to ED utilization patterns. TE increased with accuracy and produced negative values when triage performance was worse than chance. When applying the framework we found substantial gaps between theoretical and observed TE across all emergency departments, indicating significant post triage reprioritization.

**Conclusions:**

The TE framework provides the first method for directly measuring the effectiveness of triage while utilizing full ordinal priority information. TE can assess the effectiveness of initial triage and the impact of post-triage re-prioritization.

**Supplementary Information:**

The online version contains supplementary material available at 10.1186/s12874-026-02918-w.

## Introduction

Emergency departments (EDs) use triage to identify time-critical patients when immediate physician assessment is not possible [1]. The aim of triage is to reduce waiting time for time-critical patients by giving them precedence in the queue, i.e. high priority, while simultaneously assigning lower priorities to assumed non-time-critical patients so they do not unnecessarily compete for limited physician resources.

While triage has been validated using several methods using both outcome measures and expert consensus [2–4], studies looking at waiting times in relation to triage have assumed that the assigned triage priorities were correct, for example by analyzing whether higher priority cases have a shorter waiting time than those with lower priorities [5, 6]. Since the priorities are not necessarily assigned correctly, we lack evidence for how well triage systems reduce waiting times for the time-critical patients, i.e. the main purpose of triage.

Therefore, in the present study, we developed the framework Triage Effectiveness (TE), to assess this waiting time reduction. TE quantifies performance relative to two reference points: first-come-first-serve, e.g. no triage, (TE = 0%) and optimal prioritization (TE = 100%). Further, we present two complementary approaches of calculating TE: Waiting Time-based TE measures actual waiting time reduction, while Rank-based TE measures queue position improvement. Both can be calculated as Observed TE (using actual patient waiting times) or Theoretical TE (simulating strict priority adherence).

We developed the framework using patient datasets containing data from eight EDs spanning 2 years. We validated the TE framework mathematically by demonstrating alignment between simulation-based and analytical calculations. While established queueing theory methods exist for priority queues, and have been adapted to optimize triage classification under uncertainty [7] here we instead applied them for the evaluation of triage effectiveness.

## Methods

### Data sources and management

To develop and validate the TE framework, we used data from the Skåne Emergency Medicine (SEM) cohort [8], which includes all ED visits across eight hospitals in the Skåne region of Sweden during 2017 and 2018. SEM integrates data from multiple sources, including patient administration systems, electronic health records, national registries, and laboratory information systems.

Time-critical cases were identified using the Lund Outcome Set for Evaluation of Triage (LOSET) [9], a composite outcome developed through expert consensus that comprises 49 outcomes divided into two priority-specific sets: 38 outcomes for the highest (red/1) priority (requiring resuscitation team activation) and 11 outcomes for the second highest (orange/2) priority (time-critical but not requiring full resuscitation team). These outcomes include serious diagnoses (e.g., anaphylaxis, bacterial meningitis, tension pneumothorax), critical interventions (e.g., percutaneous coronary intervention, surgical airway, massive transfusion), pathological laboratory values, intensive care admissions, and mortality. To determine LOSET outcomes in the present study, we used data from multiple sources in SEM: diagnosis and intervention data from both the electronic health records (Melior™, Siemens) [10] and the Swedish National Patient Register using ICD-10 and Swedish clinical procedure codes (KVÅ) [11], blood sample data from the electronic health records, intensive care unit admissions through ward transfer records, and mortality data from the Swedish Cause of Death Register [12]. With the available SEM data, in this study we were able to implement 40 of the 49 LOSET criteria, with nine criteria excluded primarily due to lack of reliable data on medication administration in the ED and surgical scheduling.

While the LOSET framework provides a comprehensive composite outcome for identifying time-critical cases, it includes conditions with varying degrees of time sensitivity. Some conditions require immediate intervention (e.g., anaphylaxis), whereas others may tolerate longer delays without equivalent clinical deterioration. Importantly, all LOSET-defined outcomes nonetheless represent genuinely time-critical conditions; the spectrum reflects differences in the urgency of that time-criticality rather than uncertainty about its presence. Time-criticality in this study should therefore be interpreted as a spectrum above a shared clinical threshold rather than a homogeneous construct.

From SEM we also collected the initial triage priority assigned by registered nurses using the triage system RETTS [13] which is used in the region. RETTS assigns patients to one of five priority levels (1 = highest priority, 5 = lowest priority) based on clinical presentation and vital signs at arrival, with priorities 1 and 2 intended for the most urgent cases requiring immediate or rapid physician assessment. For each visit, we also captured the timestamp when the triage priority was assigned and when the patient first saw a physician, and waiting time was calculated as the interval between these time points. The pre-triage waiting was excluded as the focus was to evaluate the effect of the triage decision rather than pre-triage inefficiencies.

We included adult patient visits at any of the eight EDs who were not referred away from the ED before physician assessment, did not leave against medical advice before seeing a physician, and had chief complaints attributed to medical, surgical, or orthopedic causes in the administrative system. We did not include patients with chief complaints that indicated other medical specialities since they are handled in separate queues from the main patient flow.

At the eight EDs, there were 505,049 eligible patient visits. From this group, we excluded 41,840 patients (7.7%) with missing data. This included patients with no registered triage priority or triage time (*n* = 22,774), no recorded physician contact time (*n* = 18,684), or erroneous time points (*n* = 151). The final dataset contained 463,209 patient visits. It's worth noting that while 8.3% of the eligible overall population was excluded due to missing data, only 1.9% of time-critical cases identified by LOSET criteria were excluded for this reason, which minimizes the potential bias.

### Triage effectiveness framework

#### Waiting Time-based Triage Effectiveness (WTE)

##### **Conceptual explanation**

WTE measures how much triage reduces actual waiting times for time-critical patients compared to the average patient. If time-critical patients wait just as long as everyone else, WTE equals 0% (no effect of prioritization). If time-critical patients experience no waiting at all, WTE equals 100% (perfect prioritization). WTE can be expressed as:

1 $$WTE=1-\frac{mean\; waiting \;time \;for \;time \;critical \;patients}{mean\; waiting\; for \;all \;patients}$$

Equation 1: Formal definition of WTE

##### Calculating waiting time-based triage effectiveness

Let us consider the measured parameters:

- *W*_*tc,i*_: waiting time of time-critical patient *i*
- *W*_*i*_: waiting time of a patient *i*
- *N*_*tc*_: number of time critical patients
- *N*: number of all patients

Then, WTE is calculated as given in Eq. 2:

2 $$WTE=1-\frac{E\left[{W}_{tc,i}\right]}{E\left[{W}_{i}\right]}=1-\frac{\frac{1}{{N}_{tc}}{\sum}_{i=1}^{{N}_{tc}}{W}_{tc,i}}{\frac{1}{N}{\sum}_{i=1}^{N}{W}_{i}}$$

Equation 2: Formal expression of WTE based on measured values of waiting time.

#### Rank-based triage effectiveness (RTE)

##### Conceptual explanation

**Fig. 1:**
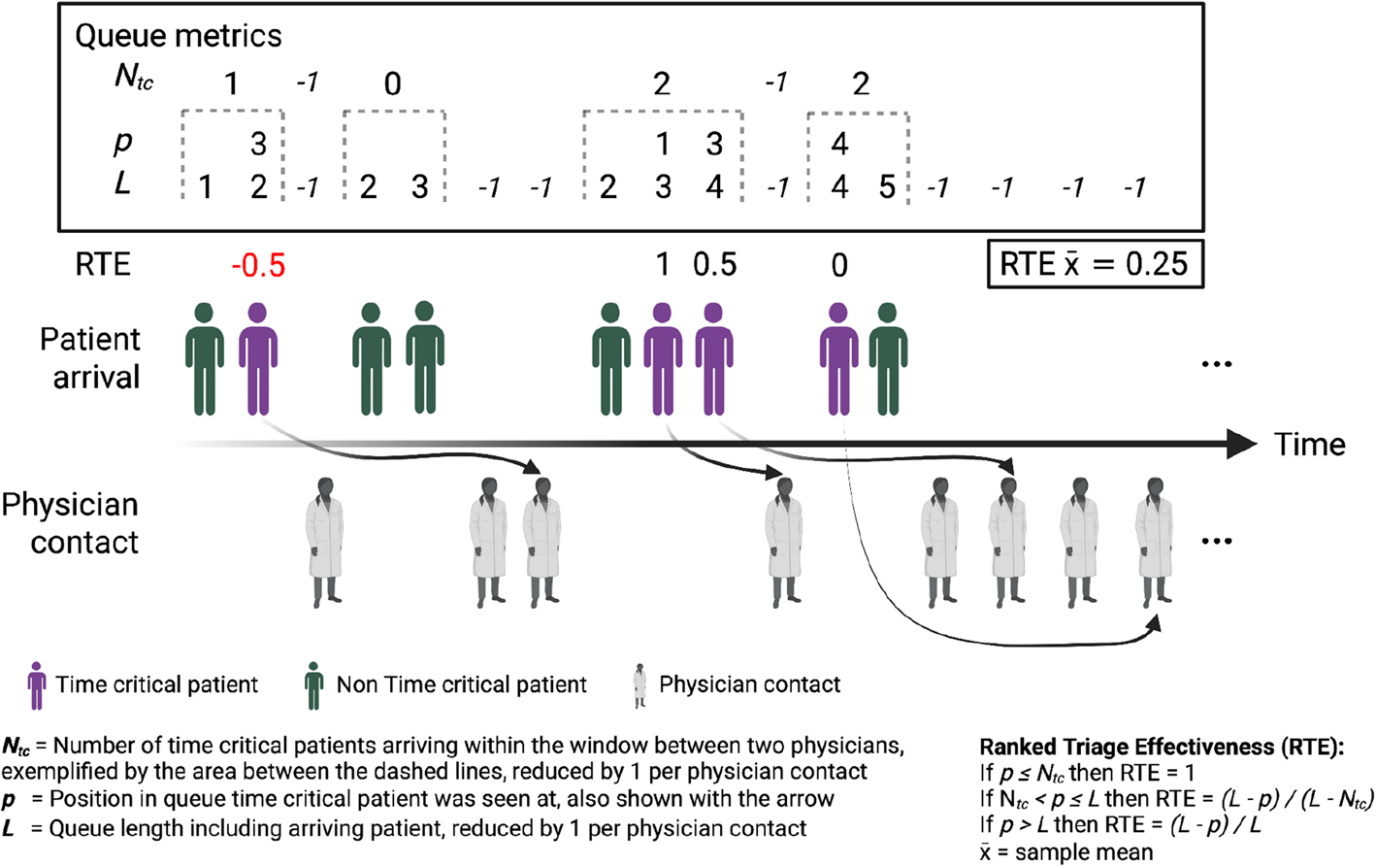
Graphical representation of how RTE is calculated showing how the queue metrics N_tc,i,_ p_tc,i_ and L_tc,i_ are calculated and how they relate to the queue structure for time critical patients. The −1’s in the L and N_tc_ row represent that these values decrease by one per physician contact

While WTE focuses on absolute waiting times, RTE measures how much closer to the front of the queue a time-critical patient is placed compared to where they would have been without triage. A value of 0% means triage provided no queue position improvement compared to a first-come-first-serve system, e.g. no triage, while a value of 100% means all time-critical patients were moved as far forward in the queue as possible, given the presence of other time-critical patients who also require prioritization. RTE evaluates queue position improvement for each individual time-critical patient and then averages these individual effectiveness scores (Fig. 1).

##### Calculating rank-based triage effectiveness

RTE calculation requires three key variables derived from the time points when the time-critical patient received the triage priority, and met the first physician:

- *L*_*tc,i*_: the queue length (L), including the time-critical patient itself, at the time of triage of patient i, which is also equivalent to their position in a first-come-first-serve system.

- *p*_*tc,i*_: the actual position in the queue (*p*) where the time-critical patient i was seen by a physician, counted from the triage time point.

- *N*_*tc,i*_: the number (*N*) of time-critical patients in the queue for patient i in the time window between two physician contacts. This decides the maximum queue position a time-critical patient could have and still be considered "perfectly prioritized". This count includes time-critical patients who arrived in previous windows but have not yet been seen by a physician, as N_*tc*_ decreases by 1 for each physician contact, similarly to *L*.

Based on the relationship between these variables, we calculate RTE using three different formulas depending on the patient's queue position:

1. Perfect triage – when the new time-critical patient is seen within the first *N*_*tc,i*_ positions:
  If *p*_*tc,i*_≤*N*_*tc,i*_ then RTE = 1

This represents cases where a time-critical patient is seen before all non time critical patients.

2. Positive RTE – when the patient is seen after an optimal position (e.g. *p*_*tc,i*_ > *N*_*tc,i*_*)* but still better, or equal, than first-come-first-serve (e.g. *p*_*tc,i*_ ≤ *L*_*tc,i*_):
  If *N*_*tc,i*_ < *p*_*tc,i*_ ≤ *L*_*tc,i*_ then RTE = *(L*_*tc,i*_*—p*_*tc,i*_*)/(L*_*tc,i*_*—N*_*tc,i*_*)*

For example, if a time-critical patient arrives in a queue that now consists of 10 patients (L_*tc,i*_ = 10), is seen 5th (*p*_*tc,i*_ = 5), and arrived together with one more time-critical patient within the queue window between physician contacts (*N*_*tc,i*_ = 2), their individual RTE = (10–5)/(10–2) = 62.5%. This shows improvement from their first-come-first-serve position (*p* = *L* = 10), where RTE would equal 0%.

3. Negative RTE – when the patient is seen even later than they would have been in first-come-first-serve:
  If *p*_*tc,i*_ > *L*_*tc,i*_ then RTE = *(L*_*tc,i*_*—p*_*tc,i*_*)/L*_*tc,i*_

For example, if a time-critical patient arrives in a queue that now consists of 10 patients (L_*tc,i*_ = 10), is seen 15th (p_*tc,i*_ = 15), their individual RTE = (10–15)/10 = −50%. This indicates the patient was prioritized lower than with a first-come-first-serve system (p = L = 10), where RTE would equal 0%.

The positive RTE formula scales relative to the achievable improvement range (L—N_tc_), while the negative RTE formula scales relative to the queue position it would receive without triage (L). This creates a natural zero point at p = L (first-come-first-serve baseline) and ensures that negative values represent a decrease proportional to the queue length at the point of triage (L), regardless of how many other time-critical patients were present.

For RTE calculations, we excluded two scenarios where meaningful prioritization evaluation was not possible: 1. Busy periods, that is periods starting with an empty queue and ending when the queue becomes empty again, where a series of only time-critical patients arrived and were processed. In this situation, all time-critical patients would have had p ≤ N_tc_ regardless of order. 2. Cases where N_tc_ = L, i.e. a queue that when the last time-critical patient arrived contained only time-critical patients, as this represents an ambiguous situation that could be interpreted as representing both perfect triage since p ≤ N_tc_ gives RTE = 1 and equivalent to a first-come-first-serve system, since p = L, RTE = 0 at the same time. Extended examples of these calculations with deeper explanations can be found in the additional file 1, Sect. 6.

After calculating RTE for each individual time-critical patient using the appropriate formula above, the overall RTE is calculated as the mean of all individual patient RTE values per ED. Formally this can be described as follows:

3 $${RTE}_{i}=\left\{\begin{array}{cc}1& \text{if } {p}_{tc,i}\le {N}_{tc,i} \\ \frac{{L}_{tc,i}-{p}_{tc,i}}{{L}_{tc,i}-{N}_{tc,i}}& \text{if } {N}_{tc,i}<{p}_{tc,i}\le {L}_{tc,i}\\ \frac{{L}_{tc,i}-{p}_{tc,i}}{{L}_{tc,i}}& \text{if } {L}_{tc,i}<{p}_{tc,i}\end{array}\right.$$

Equation 3: Piecewise RTE function for individual patients

4 $$RTE=E\left[{RTE}_{i}\right]=\frac{1}{{N}_{tc}}\sum_{i=1}^{{N}_{tc}}{RTE}_{i}$$

Equation 4: Overall RTE calculation

#### Theoretical versus observed triage effectiveness

##### Conceptual explanation

To evaluate both the quality of initial triage decisions and how well these decisions translate into actual patient care, we calculated two complementary versions of each TE measure:

*Theoretical TE* represents what TE would be if patients were seen strictly according to their initial triage priority without any subsequent reprioritizations. This isolates the quality of initial triage decisions from implementation factors. For example, if a time-critical patient is correctly assigned red/1 priority and a non-time-critical patient receives yellow/3 priority, Theoretical TE would reflect the reduced waiting time and improved queue position that results from the time-critical patient being seen first when priorities are strictly followed. Conversely, if the time-critical patient is incorrectly assigned yellow/3 priority while the non-time-critical patient receives red/1, Theoretical TE would be low because following these priorities would result in the time-critical patient waiting longer and being placed further back in the queue than the non-time-critical patient. Theoretical TE thus measures how well the triage system identifies and prioritizes time-critical patients at the point of initial assessment, capturing both the waiting time reduction (WTE) and queue position improvement (RTE) that would occur if these decisions were implemented perfectly.

*Observed TE* captures real-world performance using actual recorded waiting times or queue positions, reflecting the combined impact of triage decisions and all subsequent care processes. For example, if a time-critical patient initially assigned orange/2 priority is seen after a yellow/3 patient, the observed TE will be lower than the theoretical. Conversely, if a time-critical patient receives faster care than their initial priority indicated, observed TE may exceed theoretical. Observed TE thus captures post-triage priority changes, whether or not they involve formal reprioritization—any deviation from expected priority order is reflected in the measurement.

The difference between Theoretical TE and Observed TE, the Observed-Theoretical Gap, reveals how post-triage queue management affects patient care.

#### Deriving theoretical triage effectiveness

**Fig. 2:**
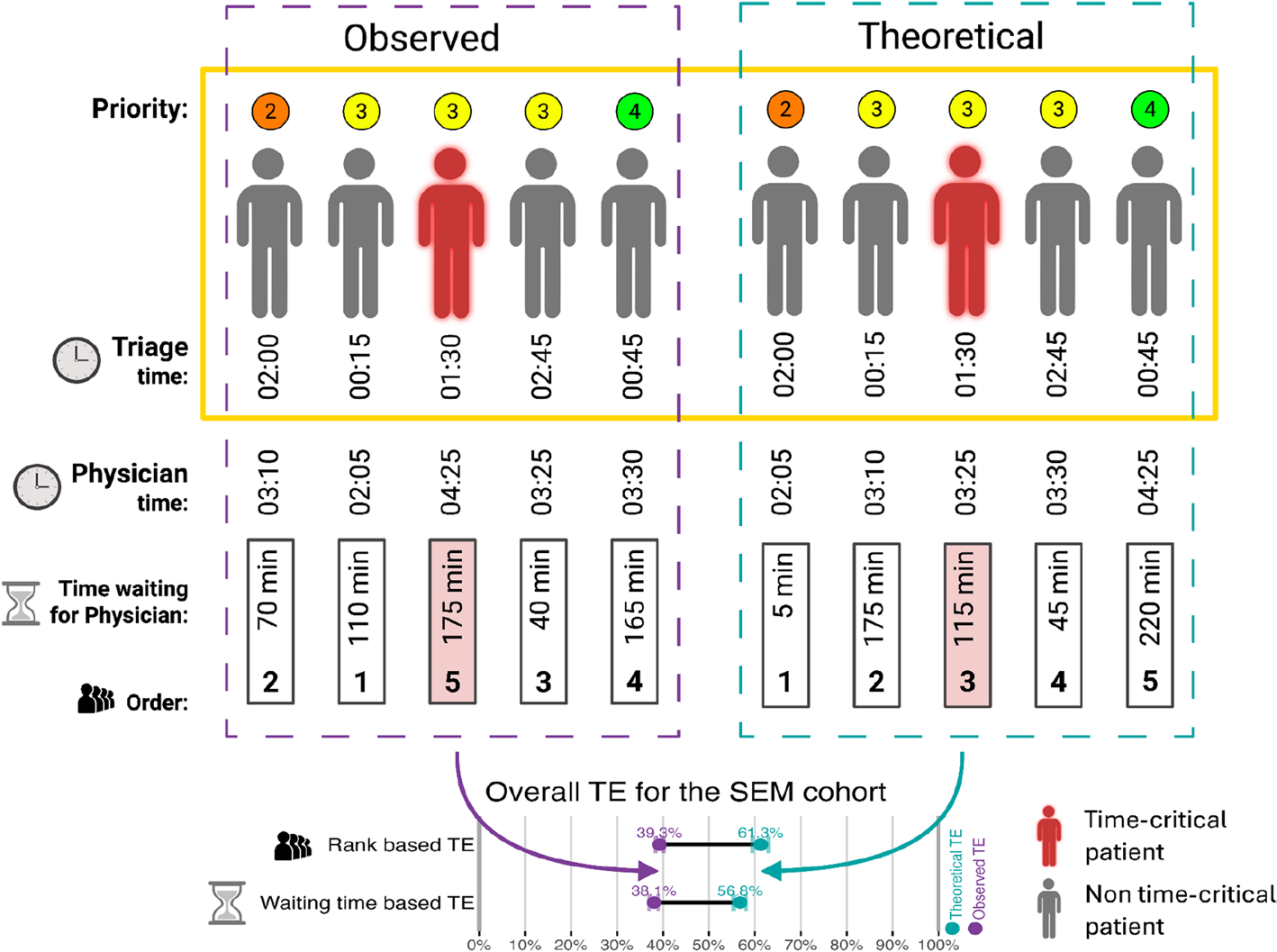
Graphical explanation of Observed (purple) and Theoretical (teal) Triage Effectiveness (TE) for the same set of five arriving ED patients. The patients are ordered first by priority (higher first) then based on arrival time (earlier first) from left to right. Priority 1 is the highest priority, and 5 is the lowest. "Triage time" is the time point where the patient receives his or her priority. “Physician time” is the time point when the patient sees the physician. “Waiting time for Physician” represents the time from the triage time until the physician time while "Order" is the order in which the patients are seen by the physicians. The gold square surrounds elements that are the same in the two calculations: priority, time-criticalness (red silhouettes vs gray silhouettes), patient characteristics and triage time. In the simulation to yield theoretical TE, the order patients are seen by physicians are set strictly by initial priority and triage time, using the original physician time slots, thereby eliminating post-triage adjustments. In the image priorities are shown with both classic triage colors and numbers

The simulation generating Theoretical TE operates in a non-preemptive manner (meaning patients are seen in strict priority order as appointments become available, without interrupting ongoing consultations) by treating physician contacts as available time slots rather than patient-specific events (Fig. 2). The model uses real-life physician contact time points rather than simulated capacity, preserving the real world temporal 24/7 patterns of ED throughput while only changing which patient is seen at each time point. This approach isolates the effect of strict priority-based queueing while maintaining a realistic throughput pattern. The detailed simulation process, implemented in R statistical software v 4.4.1 [14], can be described thematically as follows:

1. Extract a list of every date and time for all physician contacts—separate this event from the list of patients.
2. Loop through all minutes in the dataset, at each minute:
  - Add patients that were triaged at that minute to a queue, and save their priority and triage time.
  - Sort the patients based first on priority and secondly on the triage time point, forming a virtual queue.
  - If any patient saw a physician at that specific minute (based on the extracted list from step 1), remove the first patient in the virtual queue and record its waiting time.

## Deriving observed triage effectiveness

Observed TE uses actual recorded waiting times and physician contact times without any simulation. The same calculation methods apply, but the queue positions (*p*_tc,i_) and waiting times reflect real patient flow rather than simulated strict priority adherence.

### Implementation in study

For all calculations, each ED was treated as its own separate queue. When reporting overall results across all EDs, we calculated the aggregate metric as the weighted mean based on the number of time-critical cases in each ED. This approach accounted for varying patient volumes and case mix across EDs while maintaining the interpretability of the overall measure.

When calculating both RTE and WTE we treated the mean as an aggregation function rather than measures of central tendency, following aggregation theory principles as described by Beliakov et al. [15] In this framework, means are used as operators that combine individual values rather than describe data distributions.

Confidence intervals were derived through bootstrapping [16] with 2000 samples (chosen based on convergence patterns), with each iteration yielding one mean WTE/RTE value. Standard error calculations were used to extract confidence intervals from the bootstrapped distributions. We did not conduct formal statistical significance tests between individual TE values in our primary analyses. For future applications requiring statistical comparisons, we recommend bootstrap difference testing with confidence intervals extracted from the distribution of differences for the TE metric of interest.

### Queueing theory framework

#### Theoretical foundation

To validate the simulation-based TE calculations, we implemented an analytical approach building on theoretical results of M/M/1 priority queues. The M/M/1 model assumes Poisson arrival processes, exponential service times, and a single server with non-preemptive priority discipline [17]. While EDs typically operate with multiple physicians, we found that M/M/1 models have identical WTE to multi-server (M/M/c) models [18] when the system utilization remains constant, as the relative waiting time reductions between priority classes are preserved regardless of server count. A detailed proof is provided in the Additional file 1.

#### Parameter extraction from emergency department data

We calculated the total arrival rate λ as the total number of patients divided by the hours in the observation period where the given ED was open. Service rate μ was calculated as the total number of patients served divided by the duration of time when any queue existed (i.e., when patients were waiting for physician assessment). This approach assumes that physician capacity during periods without queues represents periods of genuine system downtime rather than immediate patient service. While this may slightly overestimate service rates when patients receive immediate attention without contributing waiting time to the denominator, the validation results suggest this approximation does not substantially affect the relative waiting time relationships that underlie TE calculations. System utilization ρ was then derived using the fundamental queueing relationship ρ = λ/μ.

#### M/M/1 queueing theory calculation of waiting time-based triage effectiveness

First, we calculated the prevalence of time-critical patients (prev_tc_) as the proportion of all patients meeting LOSET criteria. This splits the total arrival rate into time-critical arrivals λ_tc_ = λ × prev_tc_ and non-time-critical arrivals λ_ntc_ = λ × (1—prev_tc_). Next, we determined how time-critical and non-time-critical patients were distributed across priority levels (i.e., the triage distribution pattern). For example, the distributions might be:

**Table:** 

Priority	1	2	3	4	5	Sum
Time-critical proportion	0.45	0.25	0.15	0.10	0.05	1.00
Non-time-critical proportion	0.05	0.10	0.15	0.25	0.45	1.00

Using these distributions, we calculated the effective arrival rates for each priority level. We denoted p_tc,i_ as the proportion of all time-critical patients assigned to priority i, and p_ntc,i_ as the proportion of all non-time-critical patients assigned to priority i. We calculated the arrival rate for each priority level i as λ_i_ = λ_tc_ × p_tc,i_ + λ_ntc_ × p_ntc,i_.

We then applied the standard M/M/1 priority queueing formula [17] to calculate expected waiting times. For priority class i, the waiting time in queue is:

5 $${W}_{i}=\frac{{\rho}_{total/\mu }}{\left(1-{\sum}_{j-1}^{i-1}{\rho}_{j}\right)\left(1-{\sum}_{j=1}^{i}{\rho}_{j}\right)}$$

Equation 5: standard M/M/1 priority queueing formula for expected waiting time in queue for priority class i, where ρ_j_ represents the utilization of priority class j (λ_j_/μ), j is the summation index over priority classes, and i is the specific priority class for which we are calculating waiting time.

To calculate the average waiting time for time-critical patients, we weighted each priority's waiting time by the proportion of time-critical patients assigned to that priority: W_tc_ = Σ(p_tc_,_i_ × W_i_). The overall average waiting time was calculated as the arrival-rate-weighted average across all priorities: W_overall_ = Σ(λ_i_ × W_i_)/λ_total_.

Finally, WTE was calculated using the standard formula: WTE = 1—(W_tc_/W_overall_), representing the proportional reduction in waiting time for time-critical patients compared to the overall patient population.

### Validation

#### Queueing theory validation

We compared analytical Queueing theory TE results (e.g. Queue Theory WTE, or QTE) against simulation-based theoretical WTE calculations across the same ED data. The analytical approach was implemented using the same data preprocessing and aggregation methods as the simulation approach, including weighted means across emergency departments based on time-critical case counts to calculate the overall score.

To isolate the effect of triage distribution from system-specific factors, we also implemented QTE with standardized parameters (sQTE) by normalizing utilization to 90% and time-critical prevalence to 8% while keeping the triage distribution pattern. We used sQTE to validate RTE's independence from utilization effects.

It is important to note that analytical queueing theory methods can only validate theoretical TE calculations. Because these methods assume perfect adherence to assigned priorities, they cannot account for the post-triage reprioritization effects captured in observed TE measurements. However, since observed and theoretical TE use identical calculation methods and differ only in the data source (actual versus simulated patient flow), the validation of theoretical calculations provides confidence in the mathematical framework underlying both metrics.

#### Cross site transferability analysis

To assess the transferability of TE measurements across different hospital settings, we quantified how much TE measurements change when triage distribution patterns from one ED are applied to another ED's patient data. This analysis was done to determine whether TE metrics can be compared across different hospital contexts. That is to make sure that TE values are directly comparable across hospitals and reflect underlying triage performance rather than site-specific factors. In this context, transferability refers to the degree to which TE measurements remain stable when triage distribution patterns from one ED are applied to another ED's patient population.

The analysis was performed as follows: For each ED (the "source ED"), we extracted the triage distribution patterns, specifically, the proportion of time-critical and non time-critical patients assigned to each triage priority level. We then applied these extracted patterns to every other ED's patient data (the "target EDs"). For each source-target ED combination, we repeated this priority redistribution process 100 times using Monte Carlo simulation to account for stochastic variation in the redistribution algorithm.

For each iteration, we calculated the theoretical TE using the redistributed priorities and compared it to the source ED's original theoretical TE (calculated using the source ED's own data and triage distribution). We then calculated the mean difference across all 100 iterations for each source-target ED pair. This approach quantifies how much TE measurements change when triage distribution patterns from one hospital are applied to another hospital's patient population.

To ensure reliable estimates, we evaluated convergence of each Monte Carlo simulation by assessing the stability of running means and confidence interval across iterations. Results were summarized using mean differences and 95% confidence intervals per source-target ED pair. We visualized outcomes as heatmaps displaying the TE mean difference for each combination of original patient data and applied triage distribution.

#### Sensitivity/specificity analysis

To analyze the relationship between the TE of a triage system and its sensitivity and specificity, we dichotomized the five-level triage system into binary categories (high or low priority) and used the same technique as described in the simulation framework but with generated priorities for given values of sensitivity and specificity. In this context, sensitivity refers to the proportion of time-critical patients who are correctly assigned a high priority during triage, while specificity refers to the proportion of non-time-critical patients correctly assigned a low priority. To ensure reliable results, we preprocessed each EDs data by replicating it until achieving a minimum of 10,000 time-critical cases. In total this resulted in 4,565,143 total patient visits with 107,436 time-critical cases per sensitivity/specificity combination. Using this preprocessed data, we systematically explored combinations of sensitivity and specificity by generating triage decisions stochastically, testing every 10% increment from 0–100% for both metrics, using theoretical RTE and WTE as the outcome.

For the analytical queueing theory calculations (QTE and sQTE), we used exact triage distributions rather than stochastic generation. Since we treated this as binary triage (high priority 1–2 versus low priority 3–5), we directly set the proportions of time-critical and non-time-critical patients assigned to each priority level to match the target sensitivity and specificity values. For example, for 60% sensitivity and 80% specificity, we set 60% of time-critical patients and 20% of non-time-critical patients to high priority.

Finally, to assess the generalizability of our sensitivity/specificity analysis, we compared multilevel triage with dichotomized triage used in the heatmap. Using the same simulation framework that generated the Theoretical TE values, we calculated both Theoretical TE using all five priority levels and a binary Theoretical TE, using only high [1, 2] versus low [3–5] priority, per ED and compared the mean difference. This comparison was made to assess whether insights from our dichotomized analysis could extend to five-level triage systems.

### Software implementation

All statistical analyses in this study, including the TE calculations, queue theory calculations, simulations, and sensitivity/specificity analyses, were implemented and performed using our open-source R package 'TriEff' version 1.3 (available at: https://github.com/AndreJohanssonLund/TriEff). The package provides vectorized, computationally efficient implementations of the mathematical formulations and methodological approaches described throughout this section. The methodology presented here directly reflects the computational approaches implemented in the package. The package includes comprehensive documentation, vignettes, and an example dataset to facilitate reproducibility and extension of our work. Further details on the implementation and usage of the package can be found in the Code Availability section and Additional file 1.

## Results

### Demographics of EDs in the validation cohort

We validated TE across eight EDs representing diverse operational settings. The EDs varied substantially in size, with patient volumes ranging from 7,987 to 115,855 visits over the two-year period. System utilization ranged from 57.1% to 99.1%, demonstrating validation across different capacity constraints. Utilization measures how much of the time physicians are actively seeing patients—for example, 90% utilization means physicians spend 90% of their available time in patient consultations, with only 10% representing gaps between patients, e.g. lack of a queue. Time-critical patient prevalence varied from 5.6% to 8.7% across sites, reflecting different case mix patterns.

Triage distribution patterns were largely comparable across EDs with similar patient mixes (Table 1). Six of the eight EDs showed consistent sensitivity for identifying time-critical patients, ranging from 62.0% to 76.7% (mean 69.8%, SD 5.8%), demonstrating relatively uniform triage performance despite operational differences. However, two EDs (H and F) showed notably different patterns, with sensitivity of 31.3% and 56.1% respectively. We believe these differences stem from substantially lower proportions of priority 1 patients arriving via ambulance (29% and 44%) compared to the other facilities (68–83%). This suggests that these EDs do not receive the full scope of high-acuity ambulance cases, particularly priority 1 patients who typically present with more overt signs of critical illness. Consequently, these EDs face more challenging triage decisions with subtler presentations, resulting in lower sensitivity but higher specificity patterns (86.0% and 74.9% compared to 62.0–76.7% for the other EDs).

**Table 1:**
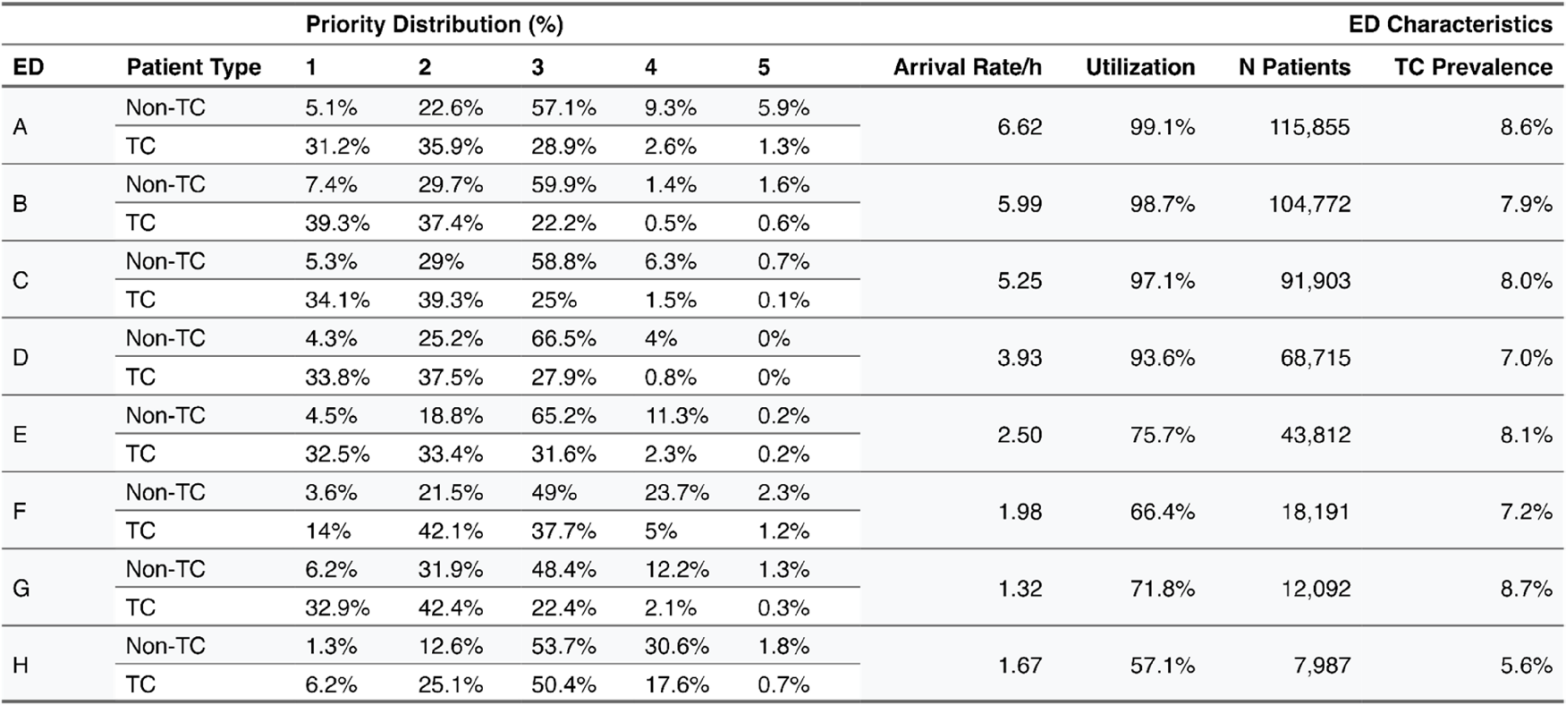
Distribution of time-critical and non-time-critical patients across triage priority levels (1-5) for each emergency department, demonstrating the priority assignment patterns used in validation analyses together with emergency department characteristics showing arrival rates, utilization, and time-critical patient prevalence across the eight validation sites, *n* = 463,327

Priority levels: 1= Most urgent, 5= Least urgent

*TC* Time-critical, defined by having a LOSET outcome

### Queueing theory validation of simulation-based TE calculations

**Fig. 3:**
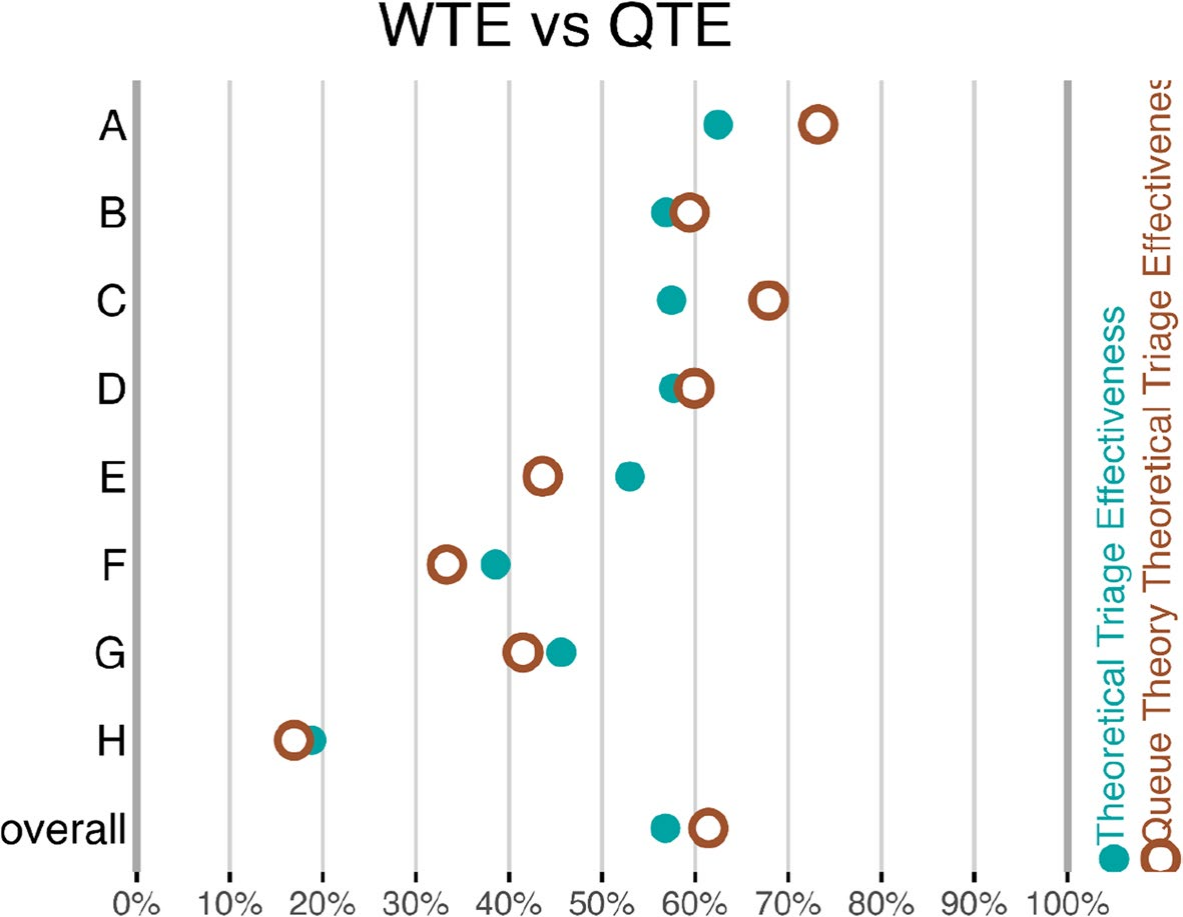
Comparison of Waiting Time-based Triage Effectiveness calculated through simulation (WTE) versus analytical queueing theory (QTE) across eight emergency departments, *n* = 463,327

The analytical queueing theory approach showed strong alignment with simulation-based TE calculations across all EDs. The mean difference between theoretical WTE and QTE was 1.10% with a standard deviation of 6.91% (Fig. 3).

**Fig. 4:**
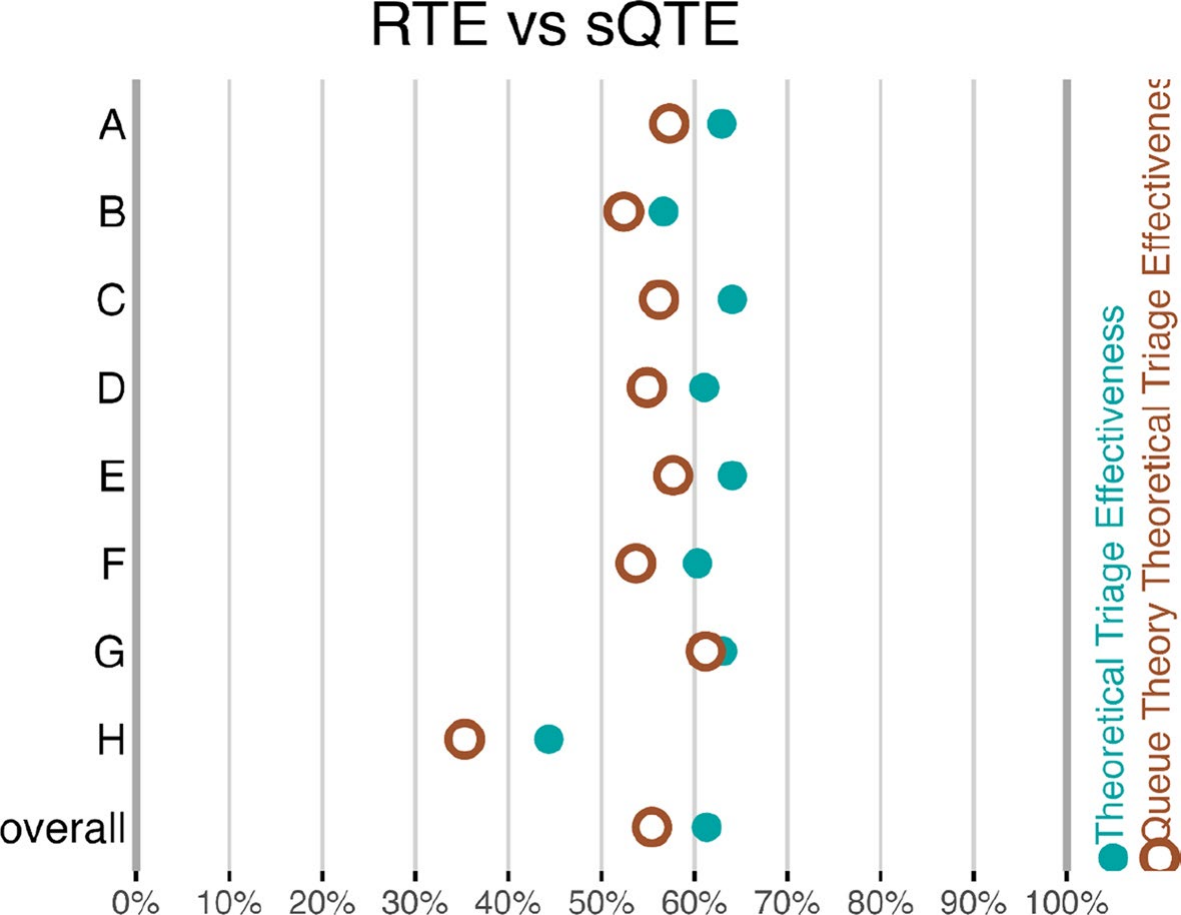
Comparison of Rank-based Triage Effectiveness (RTE) with standardized Queue Theory Effectiveness (sQTE) showing consistent offset pattern. The stable difference demonstrates RTE's normalization against utilization effects while maintaining sensitivity to triage performance, *n* = 463,327

For the comparison between RTE and sQTE, the consistent offset reflects that RTE measures queue position improvement while sQTE measures waiting time reduction under normalized conditions (Fig. 4). This stable difference pattern demonstrates RTE's independence from utilization effects that can influence waiting time-based calculations, as higher utilization leads to higher WTE values when triage distributions remain constant. Normalizing utilization to 90% in sQTE both lowers the scores for high-utilization EDs A-D while increasing scores for lower-utilization EDs E–H.

### Relationship between sensitivity, specificity and triage effectiveness

**Fig. 5:**
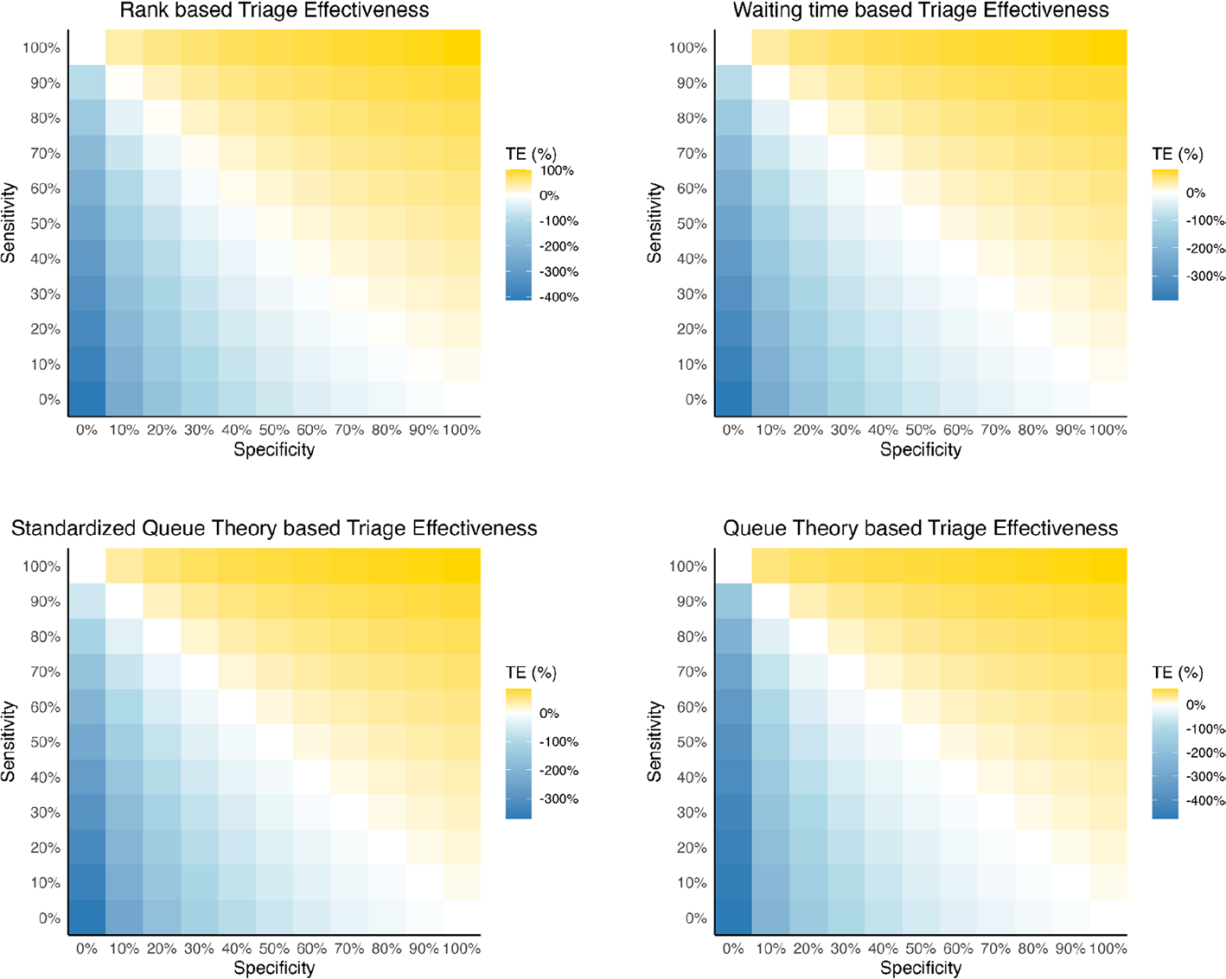
Heatmap showing mean Triage Effectiveness (TE) across sensitivity and specificity combinations, averaged across all EDs. The diagonal white line represents cases where sensitivity + specificity = 100% that nets 0% TE, below which TE becomes negative. Simulations (Rank based TE, Waiting time based TE) used data duplication to achieve 10,000 time-critical patients per ED to reduce stochastic effects (4,565,089 total visits with 107,433 time critical cases), while analytical methods used original data (463,327 visits with 36,692 time-critical cases)

The sensitivity–specificity analysis revealed the theoretical boundaries of TE performance (Fig. 5). No combination of sensitivity and specificity with a sum below 100% achieved positive TE values, aligning with classification theory where this threshold represents random performance [19]. Triage systems performing below chance (random assignment) produced negative TE values.

When sensitivity and specificity both reached 100%, RTE achieved its theoretical maximum of 100% as seen in Fig. 5, however the same is not true for WTE, QTE and sQTE where the maximum achievable value is constrained mainly by utilization, approaching 100% when utilization does (SI, Sect. 4). When simulating 100% sensitivity and 0% specificity or vice versa, all patients receive the same triage priority, creating a first-come-first-served system (i.e. no triage), resulting in 0% TE for QTE, sQTE and RTE and very close to 0% for WTE as seen in Fig. 5. The deviation from 0% in WTE occurs because the simulation uses real arrival patterns, where time-critical patients may arrive disproportionately during busy or quiet periods, causing their average wait time to differ slightly from the overall population average. Intermediate combinations showed that both sensitivity and specificity contribute to TE. When sensitivity + specificity exceeds 100%, increasing sensitivity provides greater TE improvement than equivalent increases in specificity regardless of calculation method. This relationship is reversed when sensitivity + specificity falls below 100%. In Additional file 1, Sect. 4 we also show that the magnitude of this difference increases with higher system utilization and approaches zero as utilization approaches 0%.

When comparing multilevel triage with our dichotomized evaluation, we found only modest differences in performance. The mean difference between multilevel Theoretical RTE and binary Theoretical RTE was just 10.8 percentage points (SD 7.1 percentage points) across the 8 EDs and 8.5 percentage points (SD 4.0 percentage points) for WTE. This limited difference suggests that insights from our sensitivity–specificity analysis are applicable to multi-level triage systems as well.

### Cross site transferability

**Fig. 6:**
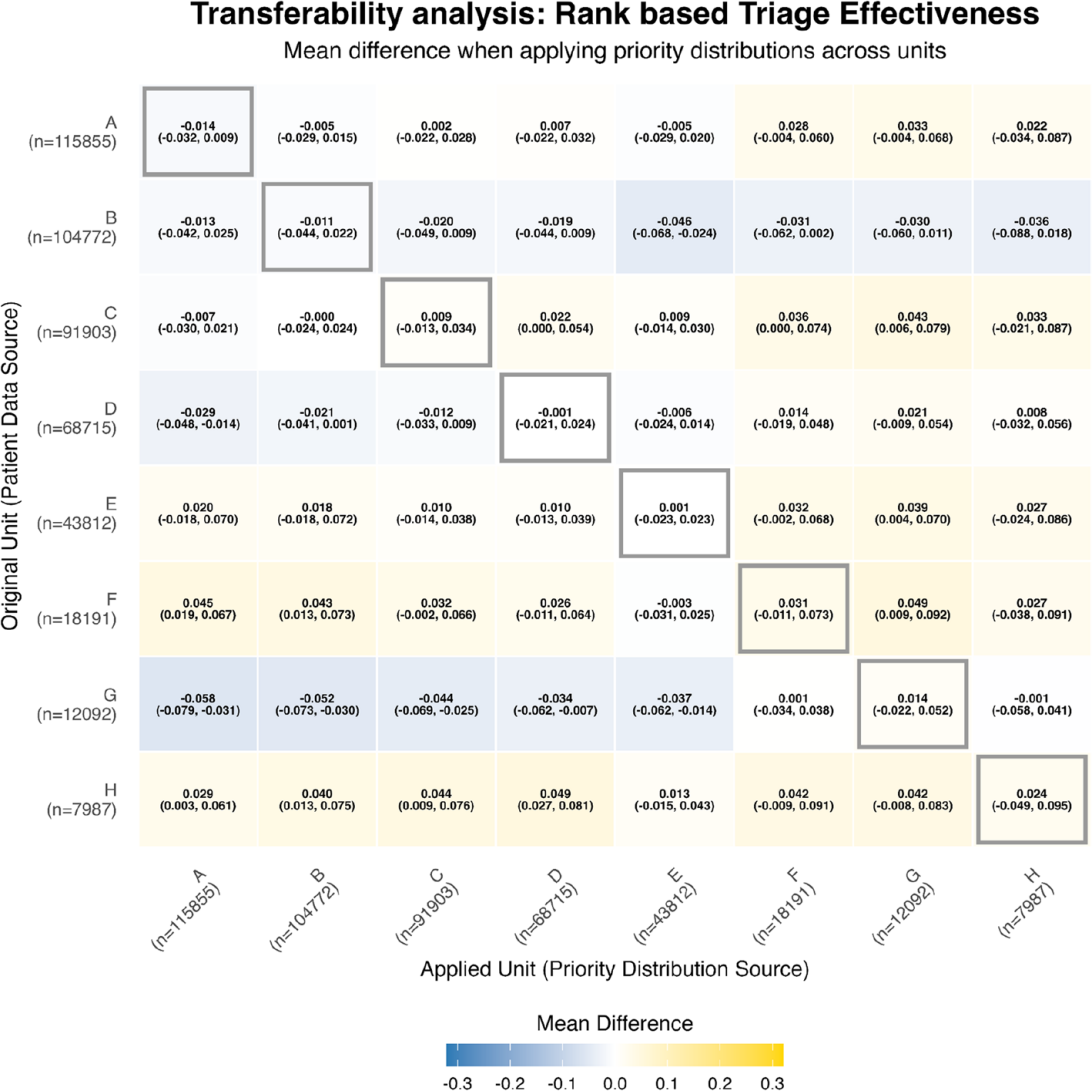
Cross-site transferability analysis of Rank-based Triage Effectiveness (RTE) showing the mean difference in TE when applying triage distributions from each ED (rows) to other EDs' patient data (columns). Results are based on 100 Monte Carlo iterations per combination. Values represent the difference from the source ED's original TE (positive values indicate the source ED's priority pattern achieved higher TE on its own patients than on the target ED's patients). The heatmap demonstrates low variability in RTE transferability across hospitals, with minimal systematic differences and slight clustering by utilization. n is shown per ED, indicating the number of patients seen over two years. Note that the coloring of the scale is adapted to be comparable to Fig. 7

The cross site transferability analysis assessed whether TE measurements reflect underlying triage performance rather than site-specific factors by testing how much TE values change when one ED's triage distribution patterns are applied to another ED's patient population. For RTE the mean difference across all ED combinations was 0.7% [95% CI: 0.6% to 0.8%], with a standard deviation of 3.4%, indicating minimal variability when applying the triage distribution of one ED to another population and suggesting that RTE captures triage distribution effects independent of site-specific factors such as utilization (Fig. 6).

**Fig. 7:**
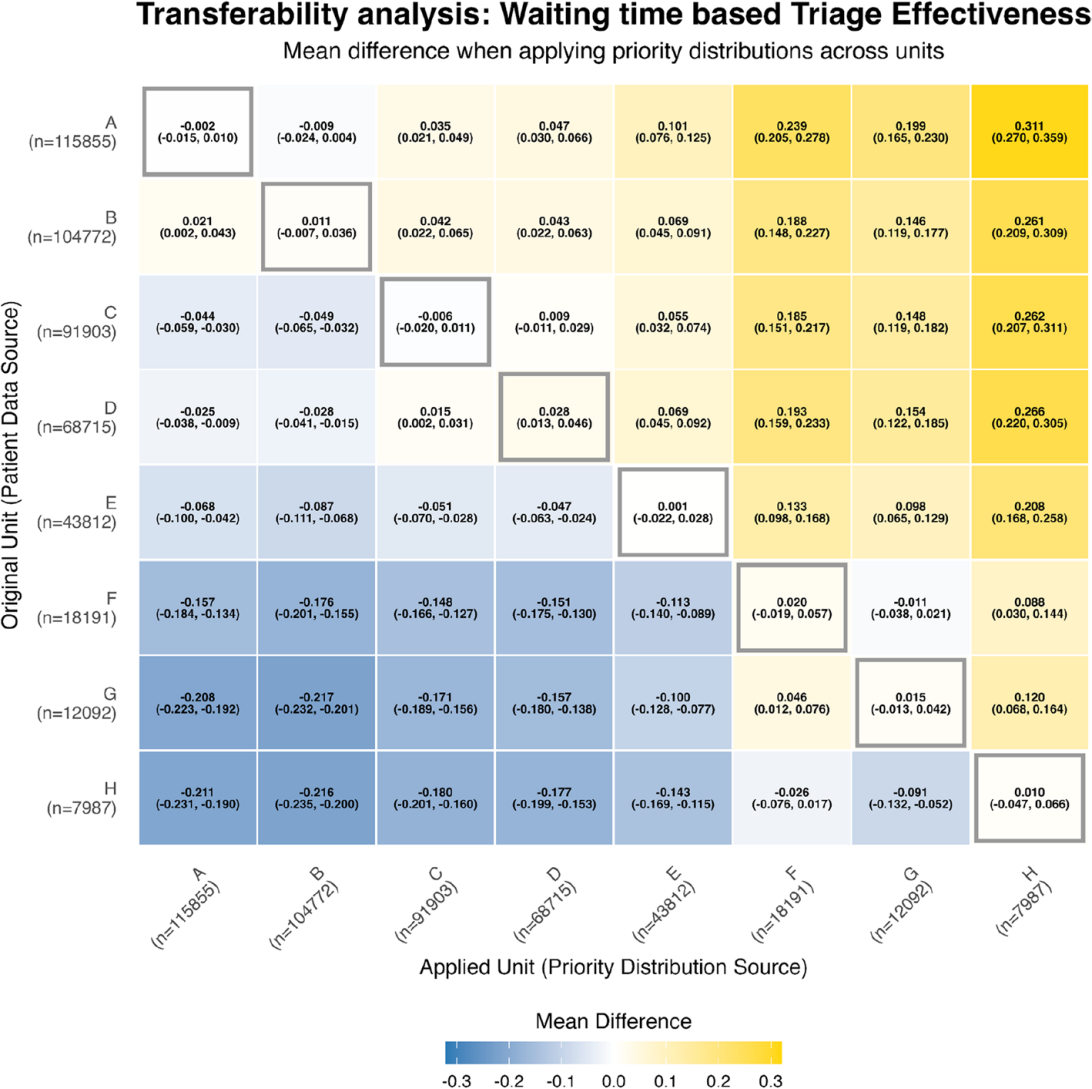
Cross-site validation of Waiting time-based Triage Effectiveness (WTE) showing the mean difference in TE when applying triage distributions from each ED (rows) to other EDs' patient data (columns). Results are based on 100 Monte Carlo iterations per combination. Values represent the difference from the source ED's original TE (positive values indicate the source ED's priority pattern achieved higher TE on its own patients than on the target ED's patients). The heatmap reveals high variability in WTE transferability, with large EDs showing low variability among themselves while small EDs demonstrate high variability both within their group and when compared to large EDs. n is shown per ED, indicating the number of patients seen over two years

In contrast, WTE showed higher variability (SD = 13.6%), with a mean difference of 1.2% [95% CI: 0.9% to 1.5%]. The transferability of WTE was strongly influenced by utilization. Large EDs (ED A, B, C and D, 99%−93% utilization), demonstrated low variability among themselves (mean difference 0.5%, SD = 3.2% [95% CI: 0.4% to 0.7%]), while small EDs with larger variation in utilization (ED E, F, G and H, 76%−57% utilization) showed higher variability within their group (mean difference 1.7%, SD = 9.8% [95% CI: 1.2% to 2.2%]) (Fig. 7). This pattern aligns with the effect from utilization demonstrated in Figs. 3 and 4, where WTE showed substantial variation with utilization while RTE remained more stable.

### Theoretical vs observed triage effectiveness

**Fig. 8:**
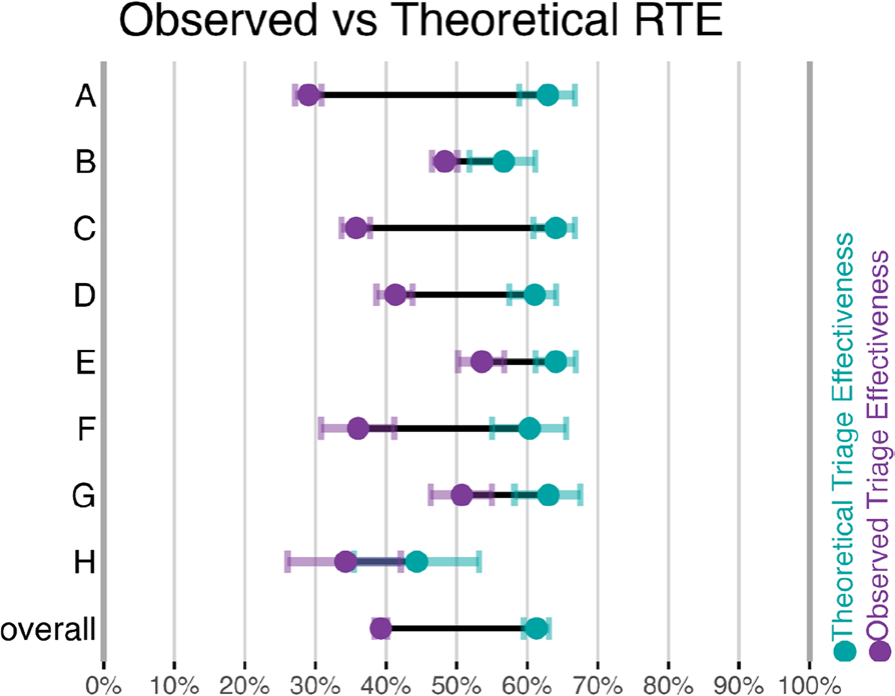
Observed Triage Effectiveness versus Theoretical Triage Effectiveness based on Ranked Triage Effectiveness (RTE) for the individual EDs in the SEM cohort showing that while the Theoretical RTE was relatively stable, representing the uniform use of one triage system, Observed RTE varied more between EDs. *n* = 463,327. Error bars represent 95% confidence intervals derived from 2000 bootstrap iterations sampled with replacement

Our previous analyses established the theoretical capabilities of triage systems, but real-world performance depends on implementation. Figure 8 presents both theoretical and observed RTE across the eight EDs, revealing the gap between the triage effectiveness achieved if initial priorities were followed (Theoretical) and actual triage effectiveness (Observed).

The observed RTE was consistently lower than theoretical RTE across all sites, with gaps ranging from −8.4% [95% CI −12.6% to −3.3%] in ED B to −33.9% [95% CI −37.8% to −30.1%] in ED A. While theoretical RTE remained relatively stable across EDs (reflecting uniform use of the same triage system), observed RTE showed substantial variation, ranging from 29.0% [95% CI 27.1% to 30.9%] in ED A to 53.6% [95% CI 50.2% to 56.7%] in ED E. This variation indicates significant differences in how well individual EDs translate triage decisions into practice.

## Discussion

In the present study, we developed and validated the concept of Triage Effectiveness (TE). To our knowledge, this is the first framework capable of evaluating a triage system's impact on time to physician in time-critical patients, providing a clinically interpretable tool.

Our analysis demonstrated that RTE and WTE showed similar patterns across EDs in both theoretical and observed measurements, though the magnitude of their values differed substantially in some settings. RTE typically showed higher values than WTE, with this difference becoming more pronounced in EDs with lower utilization rates, reflecting RTE's normalization against queue position effects rather than absolute waiting times.

Based on these findings and our cross-site validation, we recommend RTE as the preferred metric for studies comparing triage effectiveness across different EDs, as it demonstrates greater transferability. RTE also offers a conceptually more appropriate measure of triage performance because it evaluates what triage systems can actually control. While WTE measures the ultimate clinical outcome of reduced waiting times, it mixes triage decision quality with factors outside the triage system's influence. For instance, a time-critical patient triaged to priority 2 may wait 20 min due to physician unavailability when the average wait time is 40 min (assuming no other priority 2 patients are waiting), yielding a WTE contribution of 50% despite perfect triage decision-making. The same scenario would appropriately yield 100% RTE, as the patient was positioned optimally within the queue. The overall RTE of the SEM cohort was 61.3% (95% CI 59.5% to 63.1%).

We also recommend that research using this method supply sQTE as an additional reference point. Since sQTE requires only the distribution of time-critical and non-time-critical patients across priority levels rather than individual patient timestamps which WTE, RTE and QTE requires, it can serve as a standardized comparison metric even in settings with limited data granularity, ensuring broader comparability across studies with varying data availability. The overall sQTE of the SEM cohort was 55.4% (95% CI 54.5% to 56.3%).

However, sQTE has important limitations that prevent it from replacing simulation-based TE calculations. Queue theory assumes idealized conditions that do not reflect the complexity of real ED operations. More critically, sQTE cannot capture post-triage reprioritization effects, which our analysis revealed as the most substantial factor affecting patient care. The consistent gaps between theoretical and observed TE across all sites (ranging from −8.4% to −33.9%) represent clinically important phenomena that would remain invisible using analytical methods alone, yet provide the most actionable targets for ED quality improvement. While sQTE provides valuable standardized comparisons, especially for settings with limited data granularity, the simulation-based approach remains essential for identifying how well EDs translate triage decisions into practice. Both TE measures can be approximately translated to waiting times using (1 − TE) × mean waiting time. For WTE this is relatively direct. For RTE, 100% corresponds not to zero waiting time but to the minimum achievable wait given system utilization, meaning the scale runs from mean wait to lowest achievable wait rather than to zero. However, when comparing different triage systems or ED populations, the 0–100% TE scale is intended to be intuitive, and exact conversion into time units will typically not be necessary.

While the observed-theoretical gap is above interpreted as post-triage reprioritization, this can encompass a broader set of operational dynamics, including fast-track pathways for low-acuity patients, physician-driven prioritization adjustments, resource constraints, and unmeasured clinical complexity not captured at triage. Therefore, the observed-theoretical gap should not be interpreted solely as a failure to operationalise the initial triage priority, but rather as a composite indicator of downstream care processes and system-level adaptations.

### Triage effectiveness in comparison to other metrics

Zachariasse [20] established four criteria for evaluating metrics in triage assessment: Handling ordinal data, accounting for both over- and undertriage, proper scoring, and clinical utility. When examining TE against these criteria alongside existing metrics, we find that the TE offers several advantages. Unlike metrics such as sensitivity and specificity that require dichotomization, Theoretical TE can fully utilize the ordinal nature of triage priorities. As opposed to statistical measures such as Nagelkerke's R^2^, [21] the ordinal c-statistic [22], and correlation coefficients that focus on statistical associations between priority and outcome of interest, TE captures triage's clinical goal of waiting time reduction for time-critical patients.

The decision curve [23] was previously highlighted by Zachariasse [20] as having the most attractive features for triage evaluation, particularly in its ability to weight consequences of misclassification. Decision Curve Analysis evaluates trade-offs between sensitivity and specificity based on user-supplied subjective threshold probabilities that in this context represent the relative importance of under- and overtriage. In this sense it is similar to the weighted Kappa [24] but uses sensitivity and a weighted false positive rate instead of a weighted correlation metric. However both these metrics suffer from the same issue—the weighting is subjective, at best based on expert opinion, while TE inherently weighs over- and undertriage through their impact on waiting times. Like many other traditional metrics, Decision Curve Analysis also requires dichotomization of triage priorities. Finally, neither Decision Curve Analysis nor any other metric that we know of provides a direct measurement of triage's effect on the core objective of triage; waiting time reduction for time-critical cases. In contrast, TE directly measures clinical impact regardless of the number of different priorities, providing an immediately interpretable score anchored to no triage (0%) and perfect triage (100%).

While EDs often bundle other important care activities (pain management, initial diagnostic protocols, resource prediction) [25] with triage assessment, these represent distinct care dimensions that would exist independently of prioritization decisions. In this context TE specifically measures the prioritization function of triage systems rather than all activities co-occurring at triage. This approach and view is also found with most other validation methodologies discussed above [20], which similarly tries to isolate triage’s accuracy of time criticality instead of attempting to comprehensively measure every dimension of triage encounters. TE should in this context be viewed as one component of many needed to validate all care and diagnostic processes surrounding triage.

### Limitations

This study is based on several assumptions: Our theoretical TE simulation used the same physician time slots regardless of patient order, which differs from reality where changing patient order would affect physician availability. Fixed physician time slots affect RTE and WTE differently. RTE is unaffected, as it depends only on rank order within a queue window. WTE may be affected, but the impact is expected to be limited: bias only enters when multiple time-critical patients are reordered into consecutive earlier slots, since downstream slot times would shift by the difference in service times between displaced and replacing patients. Given the less than 10% prevalence of time-criticality, such consecutive reorderings are uncommon, and service time is largely unpredictable regardless time-criticalness or priority level [26], even combining treatment area, priority, complaint, and physician level explained only 45% of variance in one study [27]. QTE and sQTE are similarly expected to show small cohort-level impact for the same reason.

TE assumes a linear relationship between waiting time and adverse outcomes, treating all LOSET-defined outcomes as equally time-sensitive. In reality, the relationship between waiting time and clinical deterioration likely varies across conditions, and future research should explore weighted or stratified approaches to reflect this heterogeneity. Additionally, LOSET is applied here for the first time, and our implementation covers 40 of the 49 defined criteria. The nine excluded criteria are expected to be rare and largely co-occur with implemented outcomes, for example, inotropic support likely overlaps substantially with ICU admission, but this cannot be confirmed. The direction of any resulting bias in time-critical prevalence and TE estimates therefore remains uncertain. These limitations do not affect the validity of the TE framework itself, but do introduce uncertainty into the specific values reported, and replication using a fully implemented and independently validated outcome measure is warranted.

For the queueing theory validation, the use of M/M/1 models is a simplification. On one side, the EDs did not arrive according to a Poisson process (as shown in Additional file 1) and on the other side, we lacked data to validate that service times were exponentially distributed, as there was no way to establish a timestamp of when the physician assessment was completed. Because TE is defined as a ratio of mean waiting times, systematic approximation errors are expected to cancel between numerator and denominator, a property demonstrated formally for the M/M/c case in Additional File 1. Additional confidence comes from the convergence of simulation- and analytically-derived metrics: that WTE≈QTE and RTE≈sQTE across methodologically distinct approaches strengthens confidence in the framework without eliminating uncertainty inherent to any model-based approach. While TE can be calculated for patient subgroups (such as age or chief complaint), caution should be taken when interpreting these subgroup results causally. A detailed examination of subgroup analysis considerations for triage effectiveness measures is the subject of ongoing research. TE does not account for "fast track" systems where the ED purposefully expedites non-critical cases with the objective of reducing crowding or treating certain patient groups faster [28]. This effect could be part of the reason why Observed TE is reduced compared to Theoretical TE in our study.

Fast-track pathways are structurally at odds with TE's prioritization logic: by design they move non-time-critical patients ahead of others for reasons unrelated to clinical urgency, deflating observed TE independently of triage quality. Studies applying TE in settings with fast-track systems should therefore report whether fast-tracked patients were included, and calculate TE separately if the fast track operates as a structurally separate queue. More broadly, TE's scaling properties make it robust to differences in patient volume and crowding, and the EDs in this cohort show comparable RTE values despite widely different patient mixes [8], supporting cross-site applicability. Unmeasured structural factors beyond utilization and fast-tracking cannot be fully ruled out, and contextual description of the ED setting remains good practice when reporting TE.

Lastly, by defining arrival time as when priority was assigned rather than at ED presentation, we isolate the triage system's effect but miss the impact of delays before initial triage assessment. However, the framework can accommodate both phases separately: defining arrival as ED entry and resolution as triage completion allows TE to quantify pre-triage prioritization efficiency, while the implementation used here isolates priority triage decision performance. Combining both into a single measurement conflates their respective contributions, making it impossible to attribute TE to either process specifically. We therefore recommend analysing the two phases separately when both are of interest, which also improves cross-site comparability by making the scope of measurement explicit.

## Supplementary Information

Supplementary Material 1.

## Data Availability

The patient data used in this study are available from the authors upon reasonable request but restrictions apply for patient confidentiality reasons.
