# Supplementary material for: Triage effectiveness: a framework for quantifying the effect of emergency triage prioritization
Source: BMC Med Res Methodol. 2026 Jun 19;26:142. doi: 10.1186/s12874-026-02918-w (PMC13282852; doi:10.1186/s12874-026-02918-w)
Supplement: Supplementary file 1 — Supplementary Material 1. [file 12874_2026_2918_MOESM1_ESM.pdf]

Additional file 1

Table of Contents

1. The equivalence of WTE for M/M/1 and M/M/c models
2. Poisson arrival analysis
3. Full RTE heatmap with values
4. Heatmaps showing relationship between utilization TE, sensitivity & specificity
5. Description of the creation and validation of the Synthpop for TriEff
6. Examples of RTE calculations

1. The equivalence of WTE for M/M/1 and M/M/c models

In Section 2.3.3. we express queuing theory based WTE with the help of the M/M/1 single server queuing model. However, since an ED has several physicians working parallel, the corresponding model is a multiserver queue. Below we prove that the two models give the same WTE value, and therefore the simple M/M/1 model can be used.

We cosnider K priority queues. Recall, that λ_i is the arrival intensity of patients to priority queue i, and $\lambda_{total} = \sum \lambda_i$. Let $p_i = \frac{\lambda_i}{\lambda_{total}}$. The service intensity, measured as the intensity of patients leaving during busy periods, is μ. The load of priority queue i is $\rho_i = \lambda_i/\mu$, and $\rho_{total} = \lambda_{total}/\mu$.

According to the M/M/1 model, the average waiting time in the priority i queue is:

$$W_i = \frac{\rho_{total}}{\mu} \frac{1}{(1 - \sum_{j=1}^{i-1} \rho_j)(1 - \sum_{j=1}^{i} \rho_j)}.$$

Given $p_{tc,i}$, the probability that a TC patient is classified as priority i, the average waiting time of TC patients is:

$$W_{tc} = \sum_{i=1}^{K} p_{tc,i} W_i = \sum_{i=1}^{K} \frac{\rho_{total}}{\mu} \frac{p_{tc,i}}{(1 - \sum_{j=1}^{i-1} \rho_j)(1 - \sum_{j=1}^{i} \rho_j)},$$

while the average waiting time of all patients is:

$$W = \sum_{i=1}^{K} p_i W_i = \sum_{i=1}^{K} \frac{\rho_{total}}{\mu} \frac{p_i}{(1 - \sum_{j=1}^{i-1} \rho_j)(1 - \sum_{j=1}^{i} \rho_j)}.$$

Let us now consider the M/M/c priority queue. The service intensity when all the servers are busy is $c\mu_c$. We define the load of a priority queue as $\rho_i = \frac{\lambda_i}{c\mu}$.

In the M/M/c system, the average waiting time of queue i is [**?**]:

$$W_{i,c} = \frac{P(wait)_{M/M/c}}{c\mu} \frac{1}{(1 - \sum_{j=1}^{i-1} \rho_j)(1 - \sum_{j=1}^{i} \rho_j)},$$

where $P(wait)_{M/M/c}$ is the probability that an arriving patient needs to wait. W_{tc} and W is then calculated as for M/M/1 systems.

Note that WTE contains the ratio of the waiting time values W_{tc} and W. Therefore, for $\mu = c\mu_c$ and identical arrival intensities the WTE values are the same under the M/M/1 and the M/M/c models.

This has the practical consequence that the WTE value can be calculated with the M/M/1 model, without knowing the exact number of servers, that is, the number of physicians, an by estimating the service intensity μ by observing the inter-departure time of the patients in busy periods.

2. Poisson arrival analysis

Poisson Arrival Process Analysis by Emergency Department			
**ED Unit**	**Arrival Rate (λ)**	**SD of λ**	**Index of Dispersion (Variance/Mean)**
A	6.62	3.86	2.25
B	5.99	3.86	2.49
C	5.25	3.41	2.21
D	3.93	2.73	1.90
E	2.50	1.96	1.53
F	1.98	1.59	1.27
G	1.32	1.13	0.96
H	1.67	1.03	0.64

Suppl. Table 1: Poisson arrival process validation across emergency departments. Analysis of hourly arrival patterns during operating hours for each emergency department to validate the Poisson arrival assumption used in M/M/1 queueing theory calculations. The Index of Dispersion represents the variance-to-mean ratio, where a value of 1.0 indicates perfect adherence to the assumption of a Poisson arrival process. Values greater than 1.0 indicate overdispersion (greater variability than expected under Poisson).

3. Full RTE heatmap with values

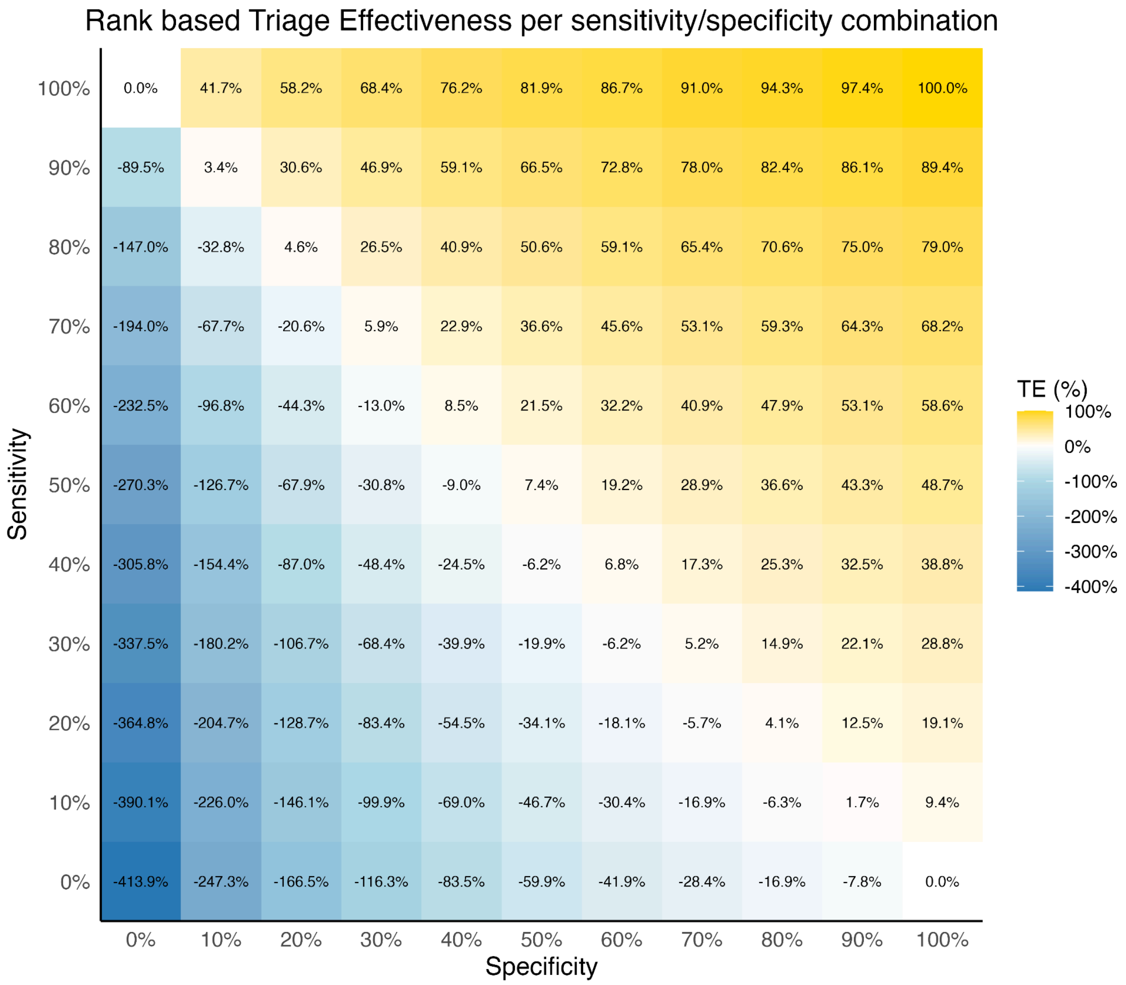

Figure s1: Heatmap showing mean Ranked Triage Effectiveness (RTE) across EDs with the data for each ED duplicated to achieve at least 10,000 time-critical patients. The diagonal line from the upper left corner to the lower right represents values where sensitivity + specificity = 100%. Due to duplication, each heatmap cell contains 4,565,089 patient visits with 107,433 time critical cases each.

4. Heatmaps showing relationship between utilization TE, sensitivity & specificity

(see next page)

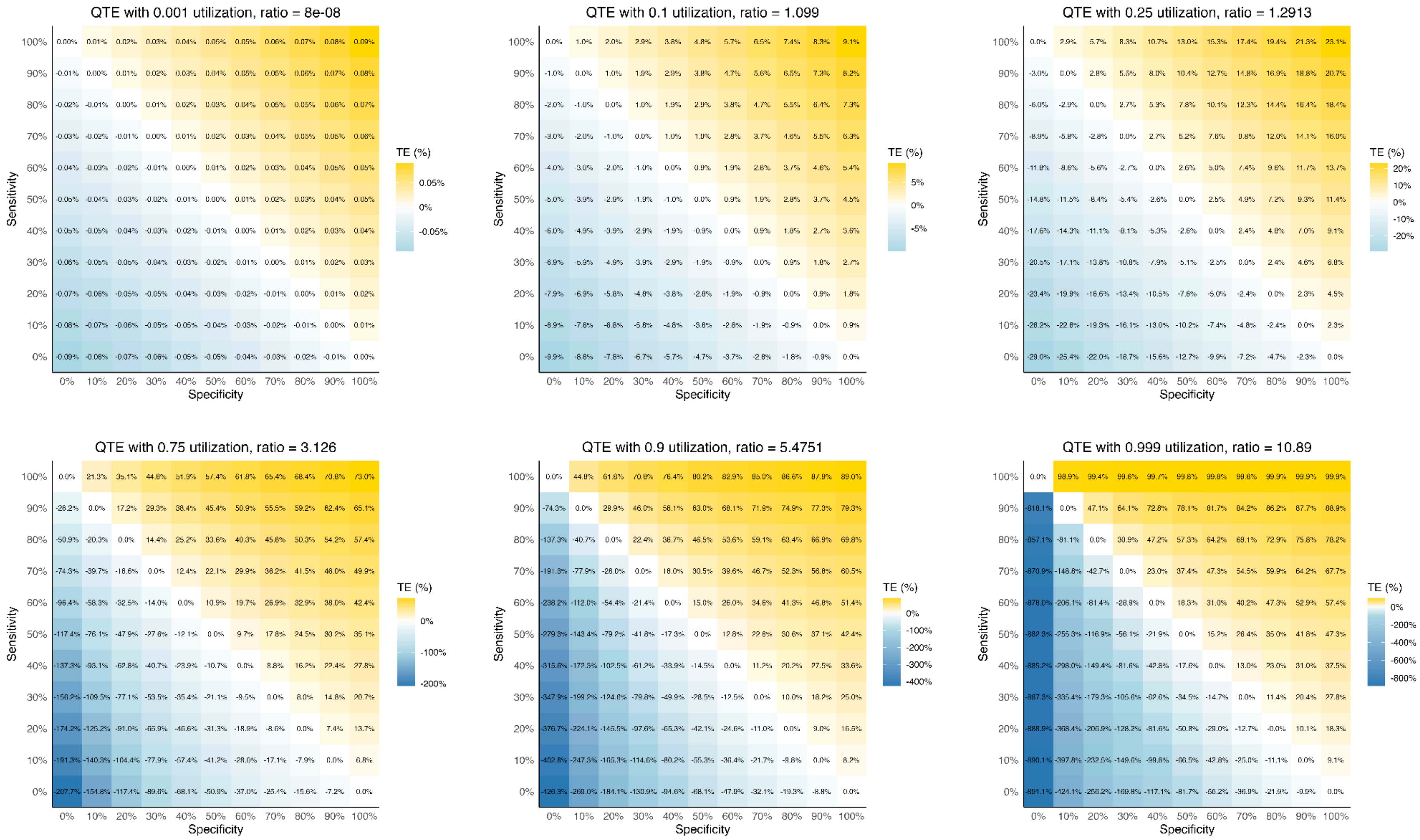
QTE with 0.001 utilization, ratio = 8e-08
QTE with 0.1 utilization, ratio = 1.099
QTE with 0.25 utilization, ratio = 1.2913
QTE with 0.75 utilization, ratio = 3.126
QTE with 0.9 utilization, ratio = 5.4751
QTE with 0.999 utilization, ratio = 10.89
Sensitivity
Specificity
TE (%)

Figure s2: Heatmaps showing the relationship between sensitivity, specificity, and utilization using Queueing Theory-based Triage Effectiveness (QTE) with time-critical prevalence set to 10%. For each heatmap, the title shows the ratio between TE at 100% sensitivity/10% specificity and TE at 10% sensitivity/100% specificity. A higher ratio indicates that sensitivity contributes more to TE than specificity when sensitivity + specificity > 100%. This relationship reverses on the negative side of the TE scale (e.g. when sensitivity + specificity < 100%), where increased utilization makes TE more responsive to specificity than sensitivity. The figure demonstrates that this effect approaches zero as utilization approaches 0%. The figure also shows that maximum achievable TE (i.e., at 100% sensitivity, 100% specificity) is constrained by utilization, approaching 0% as utilization nears 0% and approaching 100% as utilization nears 100%.

5. Description synthpop generated for TriEff package

Overview

To enable testing and validation of the TriEff statistical package, we generated a synthetic dataset based on emergency department visits across two of the hospitals in the Skåne Emergency Medicine (SEM) cohort. The synthetic dataset preserves statistical relationships and patterns from the complete SEM database while containing no data from real patients.

Methods

Data Preprocessing

Starting with the cleaned dataset from the original study, we performed additional preprocessing steps specific to synthetic data generation. Chief complaints were translated to English and systematically recoded into clinically meaningful categories (e.g., various pain-related complaints were grouped, trauma types were consolidated). Chief complaints with fewer than 300 cases were excluded to ensure robust synthetic data generation. This preprocessing removed 4.6% of the total patient population while significantly improving the quality of synthetic data generation.

Temporal variables were standardized as proportional measures to facilitate synthesis across different hospital flow patterns. Time to physician was expressed as a fraction of total ED stay, and priority assignment time as a fraction between arrival and physician contact. This approach ensured realistic temporal relationships in the synthetic data without requiring the synthesis algorithm to generate exact time points. Finally we filtered and used only two

of the EDs C and E for the first year of the cohort to bring down the data size but still keep temporal variations during one year.

Synthetic Data Generation

We used the synthpop R package to generate synthetic data, implementing a grouped approach based on chief complaints to better preserve the relationships between variables critical for triage effectiveness calculations. We generated synthetic data following a logical sequence that mimics the actual patient journey through the ED. This began with patient characteristics (age, gender), followed by arrival circumstances, clinical assessments (including triage priority and unit assignment), and concluded with process times and outcomes. To ensure complete de-identification, we applied a jittering algorithm to eliminate any rows that remained identical to the original data across key variables. The algorithm identified 0 such rows. The complete variable sequence and detailed implementation are available in the supplementary code.

Validation

The synthetic dataset underwent validation to ensure it maintained key statistical properties of the original data while preserving patient privacy. We compared univariate distributions for all variables and assessed the preservation of critical relationships between variables.

Key variable comparisons between the original and synthetic datasets are shown in Suppl Table 1. The synthetic data closely matched the original data's priority level distribution, with differences of less than 1 percentage point across most variables. Age distributions were also well-preserved, with nearly identical means and standard deviations.

Comparison of Original and Synthetic Data by Unit											
	Demographics				Priority Distribution					Outcome	
Dataset	Age (SD)[ab]	Male	LOSET+	Ambulance	1	2	3	4	5	Admitted	Waiting time (SD)[ac]
**C**											
Original	57.7 (21.9)	48.6%	6.9%	28.8%	6.4%	25.9%	63.8%	3.9%	0.0%	36.0%	87.3 (95.2)
Synthetic	58.0 (21.7)	48.5%	6.9%	28.7%	5.8%	25.9%	64.0%	4.3%	0.0%	35.9%	87.9 (95.2)
**E**											
Original	59.4 (20.7)	48.1%	8.3%	26.4%	6.3%	19.2%	62.4%	11.9%	0.2%	28.1%	49.7 (80.6)
Synthetic	59.6 (20.8)	47.6%	7.7%	26.5%	5.7%	20.4%	62.7%	11.0%	0.2%	28.5%	51.3 (85.8)

[a] Values represent means
[b] Measured in years
[c] Measured in minutes

Suppl. Table 2: Comparison of key variables between the original data in SEM (named Original, n = 55,721) and the synthpop created based on the same population (named Synthetic, n = 53,265).

The triage effectiveness metrics for both datasets are compared in Figure s3 and s4. While the synthetic data shows some differences in the Observed TE, it maintains sufficient similarity in the relative performance patterns across *complaint categories* to serve as an effective test dataset for the TriEff package.

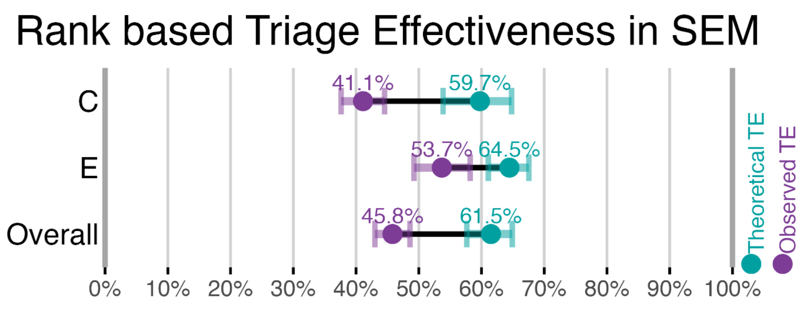

Figure s3: Rank based Triage Effectiveness (RTE) for the SEM, separated into EDs categories, this is identical to Figure 2 in the main manuscript. n = 55,721. Error bars represent 95% confidence intervals derived from 2000 bootstrap iterations.

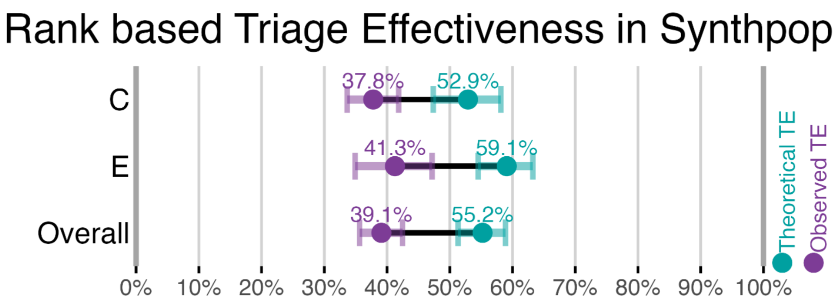

Figure s4: Rank based Triage Effectiveness (RTE) for the Synthpop, separated into EDs categories. n = 53,265. Error bars represent 95% confidence intervals derived from 2000 bootstrap iterations.

Limitations

This synthetic dataset is intended for testing the TriEff statistical package and should not be considered equivalent to the original SEM data. While it preserves many important statistical relationships, some metrics show notable differences from the original data. These differences do not impact its utility for package testing but should be considered when using the synthetic data for other purposes.

Data Availability

The synthetic dataset is included in the open-source R package 'TriEff' version 1.3 (available at: https://github.com/AndreJohanssonLund/TriEff).

6. Extended examples of RTE calculations

This section is provided as a guidance on how RTE is calculated with examples and explanations for each metrics after the examples. This can be seen as an explanatory companion to section 2.2.2. in the main article.

Short queue (L = 4, N_{tc} = 1)

A time-critical patient arrives at an ED where three other patients are already waiting, and no other time-critical patient is present. L = 4, N_{tc} = 1.

If the patient is seen first (p = 1), they have been maximally prioritized. Their individual RTE = 1, or 100%.

If the patient is seen third (p = 3), they were prioritized ahead of first-come-first-serve but not ideally. Their individual RTE = (4 − 3) / (4 − 1) = 1/3 = 33%.

If the patient is seen fifth (p = 5), they were seen after patients who arrived after them, faring worse than in a first-come-first-serve queue. Their individual RTE = (4 − 5) / 4 = −25%.

Medium queue (L = 10, N_{tc} = 2)

A time-critical patient arrives at an ED where nine other patients are already waiting. Within the same queue window, that is, between the same two consecutive physician contacts, one other time-critical patient also arrived. L = 10, N_{tc} = 2.

If the patient is seen second (p = 2), It was seen in a position that would be validly one of two of the best positions it could have been seen at. Their individual RTE = 1, or 100%. However it can be noted that a non time-critical patient could have been seen at p = 1, this would then

affect the other time-critical patient arriving in the same windows individual RTE, since it was not seen first or second.

Said timecritical patient could for example be seen sixth ($p = 6$), they were prioritized ahead of first-come-first-serve but not to the achievable ideal. Their individual RTE = (10 − 6) / (10 − 2) = 4/8 = 50%.

If any of the time-critical patients were seen twelfth ($p = 12$), they were seen after patients who arrived after them. Their individual RTE = (10 − 12) / 10 = −20%.

Long queue ($L = 25$, $N_{tc} = 3$)

A time-critical patient arrives at an ED where twenty-four other patients are waiting. Within the same queue window (e.g. between two physician contacts), two other time-critical patients also arrived, making three time-critical patients competing for prioritization within this window. $L = 25$, $N_{tc} = 3$.

If the first of these patients is seen third ($p = 3$), they were placed in one of the three positions that would constitute maximum prioritization. Their individual RTE = 1, or 100%. However, as with the medium queue example, we do not know what $p = 1$ and $p = 2$ were: if any non-critical patient occupied those positions, the remaining time-critical patients could not all achieve RTE = 100%.

One of the remaining time-critical patients could for example be seen tenth ($p = 10$), reflecting meaningful prioritization but with substantial room for improvement. Their individual RTE = (25 − 10) / (25 − 3) = 15/22 = 68%.

If the third time-critical patient were seen twenty-eighth ($p = 28$), they were seen after patients who arrived after them. Their individual RTE = (25 − 28) / 25 = −12%.

Carry-over of N_{tc}.

To illustrate how N_{tc} evolves across physician contacts, consider a scenario where three time-critical patients were present in an earlier queue window with L = 30, N_{tc} = 3 for those patients. Two physician contacts then occur, reducing both L and N_{tc} by one each, regardless of whether the patients seen were time-critical or not. After the two physician contacts N_{tc} = 1. This is a key distinction: N_{tc} does not track how many time-critical patients remain in the queue, but rather how many are competing for the optimal front positions in the current window, meaning that in our example, only one of the original three time-critical patients can still claim an optimal position. Returning to our example, four non-critical patients arrive in the interim, and one new time-critical patient, now containing twenty-seven others. L = 28, N_{tc} = 2: the one remaining carry-over time-critical patient plus the new arrival, both now competing for the first two positions.

If this new patient is seen second (p = 2), they are placed in one of the two positions that constitute optimal prioritization. Their individual RTE = 1, or 100%. Whether the carry-over time-critical patient also achieved an optimal position depends on what occupied p = 1.

If the new patient is seen eighth (p = 8), they were prioritized ahead of first-come-first-serve but not to the achievable ideal. Their individual RTE = (28 − 8) / (28 − 2) = 20/26 = 77%.

If the new patient is seen thirtieth (p = 30), they were seen after patients who arrived after them. Their individual RTE = (28 − 30) / 28 = −7%.

Exclusion scenarios in RTE calculations

RTE calculations exclude queue windows where the number of time-critical patients equals the total queue length ($N_{tc} = L$), meaning all patients waiting at the time of the last time-critical arrival are themselves time-critical. This scenario renders the metric uninterpretable, and including it would introduce a systematic error depending on how such windows are handled.

The problem is that the same queue window can simultaneously satisfy the conditions for both perfect triage and first-come-first-serve. Every patient occupies a position $p \leq N_{tc}$, which by definition yields RTE = 100%, yet every patient can at the same time also be seen in exactly the order they arrived at, which is indistinguishable from no prioritization occurring. There is no logical basis for choosing one interpretation over the other. The likeliest occurrence of this happening is when $L = N_{tc} = p = 1$. That is, one time-critical patient arrives, and gets care immediately, which exemplifies that we cannot derive if the immediate care was due to the triage decision at all.

If such windows are scored as RTE = 100%, overall triage effectiveness is inflated, a queue of only time-critical patients would contribute maximum scores regardless of whether any prioritization effort was made. Conversely, if scored as RTE = 0%, performance is artificially deflated, a triage system that successfully concentrated time-critical patients into the same window would be penalized for doing so. Either way, the measurement no longer reflects the quality of triage decisions. Excluding these windows avoids this ambiguity and ensures that RTE only captures situations where meaningful prioritization evaluation is possible.
